# Role of the tumor microenvironment in PD-L1/PD-1-mediated tumor immune escape

**DOI:** 10.1186/s12943-018-0928-4

**Published:** 2019-01-15

**Authors:** Xianjie Jiang, Jie Wang, Xiangying Deng, Fang Xiong, Junshang Ge, Bo Xiang, Xu Wu, Jian Ma, Ming Zhou, Xiaoling Li, Yong Li, Guiyuan Li, Wei Xiong, Can Guo, Zhaoyang Zeng

**Affiliations:** 1 NHC Key Laboratory of Carcinogenesis (Central South University) and Hunan Key Laboratory of Translational Radiation Oncology, Hunan Cancer Hospital and the Affiliated Cancer Hospital of Xiangya School of Medicine, Central South University, Changsha, 410013 Hunan China; 2 The Key Laboratory of Carcinogenesis and Cancer Invasion of the Chinese Ministry of Education, Cancer Research Institute and School of Basic Medical Science, Central South University, Changsha, 410078 China; 3 Hunan Key Laboratory of Nonresolving Inflammation and Cancer, Disease Genome Research Center, The Third Xiangya Hospital, Central South University, Changsha, 410013 Hunan China; 4 Department of Chemistry, University of North Dakota, Grand Forks, North Dakota 58202 USA; 5 Department of Cancer Biology, Lerner Research Institute, Cleveland Clinic, Cleveland, OH 44195 USA

**Keywords:** Tumor immune escape, PD-L1, PD-1, Tumor microenvironment, Inflammatory factor

## Abstract

Tumor immune escape is an important strategy of tumor survival. There are many mechanisms of tumor immune escape, including immunosuppression, which has become a research hotspot in recent years. The programmed death ligand-1/programmed death-1 (PD-L1/PD-1) signaling pathway is an important component of tumor immunosuppression, which can inhibit the activation of T lymphocytes and enhance the immune tolerance of tumor cells, thereby achieving tumor immune escape. Therefore, targeting the PD-L1/PD-1 pathway is an attractive strategy for cancer treatment; however, the therapeutic effectiveness of PD-L1/PD-1 remains poor. This situation requires gaining a deeper understanding of the complex and varied molecular mechanisms and factors driving the expression and activation of the PD-L1/PD-1 signaling pathway. In this review, we summarize the regulation mechanisms of the PD-L1/PD-1 signaling pathway in the tumor microenvironment and their roles in mediating tumor escape. Overall, the evidence accumulated to date suggests that induction of PD-L1 by inflammatory factors in the tumor microenvironment may be one of the most important factors affecting the therapeutic efficiency of PD-L1/PD-1 blocking.

## Background

Tumor immune escape refers to the phenomenon by which tumor cells can grow and metastasize by avoiding recognition and attack by the immune system through various mechanisms, which is an important strategy for tumor survival and development [1].The are many inducible factors of tumor immune escape, including the low immunogenicity of tumor cells, recognition of tumor-specific antibodies as autoantigens, tumor surface antigen modulation, tumor-induced exemption regions, and tumor-induced immunosuppression, the latter of which has been the most extensively studied mechanism to date. Tumor-induced immunosuppression operates in two main ways. The first occurs by inducing immunosuppressive cells to accumulate around the tumor and secrete immunosuppressive factors, which inactivate cytolytic T lymphocytes (CTL) to decrease the immune tolerance of tumor cells, such as regulatory T cells (Treg cells) [2, 3], myeloid-derived suppressor cells (MDSCs) [4], dendritic cells (DCs) [5], and M2 macrophages [6, 7].The second mechanism of immunosuppression involves induction of the expression of immunosuppressive molecules or their receptors, including programmed death-ligand 1/programmed death-1 (PD-L1/PD-1), galectin-9/TIM-3, IDO1, LAG-3, and CTLA4, which are known as the immune checkpoints and can inhibit the activation of effector T lymphocytes, ultimately leading to tumor immune escape. Thus, blocking these immune checkpoints has become an important direction of immunotherapy in recent years to eliminate the immune suppression and restore immune system function. Among these immune checkpoint blockers, PD-L1/PD-L1 antagonists account for the largest proportion of drugs approved by the FDA in recent years and are currently in clinical trials.

PD-1, also known as cluster of differentiation 279 (CD279), has attracted a substantial amount of attention in the field of cancer research in recent years. PD-1 was originally cloned from a drug-treated mouse hybridoma and hematopoietic progenitor cell lines in an apoptotic state by subtractive hybridization in 1992, and was considered to be mainly involved in the process of cell apoptosis, from which its name is derived [8]. Human PD-1 is encoded by the *PDCD1* gene, located on chromosome 2q37, which is a type I transmembrane protein composed of 288 amino acid residues, belonging to the immunoglobulin CD28 family. PD-1 is expressed in a wide range of immune cells, including peripherally activated T cells, B cells, monocytes, natural killer (NK) cells, and certain DCs. Weaker PD-1 expression has also been detected on the surface of immature T cells and B cells located in the thymus and bone marrow during specific developmental stages [9, 10]. When binding to its ligand, PD-1 can activate intracellular signaling pathways and inhibit the activation of immune cells, thereby reducing the secretion of antibodies and cytokines by immune cells to even exhaust the immune cell and thus maintain immune system homeostasis. PD-L1 (B7-H1 or CD274) was the first ligand of PD-1 discovered [11], which belongs to the B7 family and is located on human chromosome 9 p24.2. Its amino acid structure is similar to that of PD-1. PD-L1 is widely expressed. In addition to lymphocytes, PD-L1 is also widely expressed in non-blood cells such as in lung, vascular endothelium, reticular fibroblasts, non-parenchymal liver cells, mesenchymal stem cells, islet cells, astrocytes, neuronal cells, and keratinocytes [9, 12, 13]. In addition, PD-L1 also shows abnormally high expression in tumor cells, which is considered the main factor responsible for promoting the ability of tumor immune escape [14–17].

However, the therapeutic effect of a PD-1/PD-L1 antagonist against solid tumors is currently not satisfactory. In PD-L1-positive metastatic melanoma or lung cancer, the effective rate of anti-PD-L1 antagonists is only 40–50%. In colorectal cancer, although the PD-L1-positive rate is 40–50%, anti-PD-1 or anti-PD-L1 drugs show very low efficacy [18]. This poor treatment response, in addition to the high variation of genetic mutations among individuals, may also be related to the complex microenvironment of tumors. The role of the tumor microenvironment in tumor growth and metastasis has long been recognized. Recent studies have also shown that many cytokines and tumor-derived exosomes in the tumor microenvironment can induce the expression of PD-L1 and promote tumor immune escape. This review provides a summary of recent research progress toward understanding the molecular mechanism of PD-L1/PD-1 in tumor immune escape, and the regulation of PD-1 and PD-L1 in the tumor microenvironment. This research progress and indication of remaining questions can help to better understand the tumor immune escape mechanism toward developing more effective immunotherapies for cancer patients.

## Tumor microenvironment

**Fig. 1:**
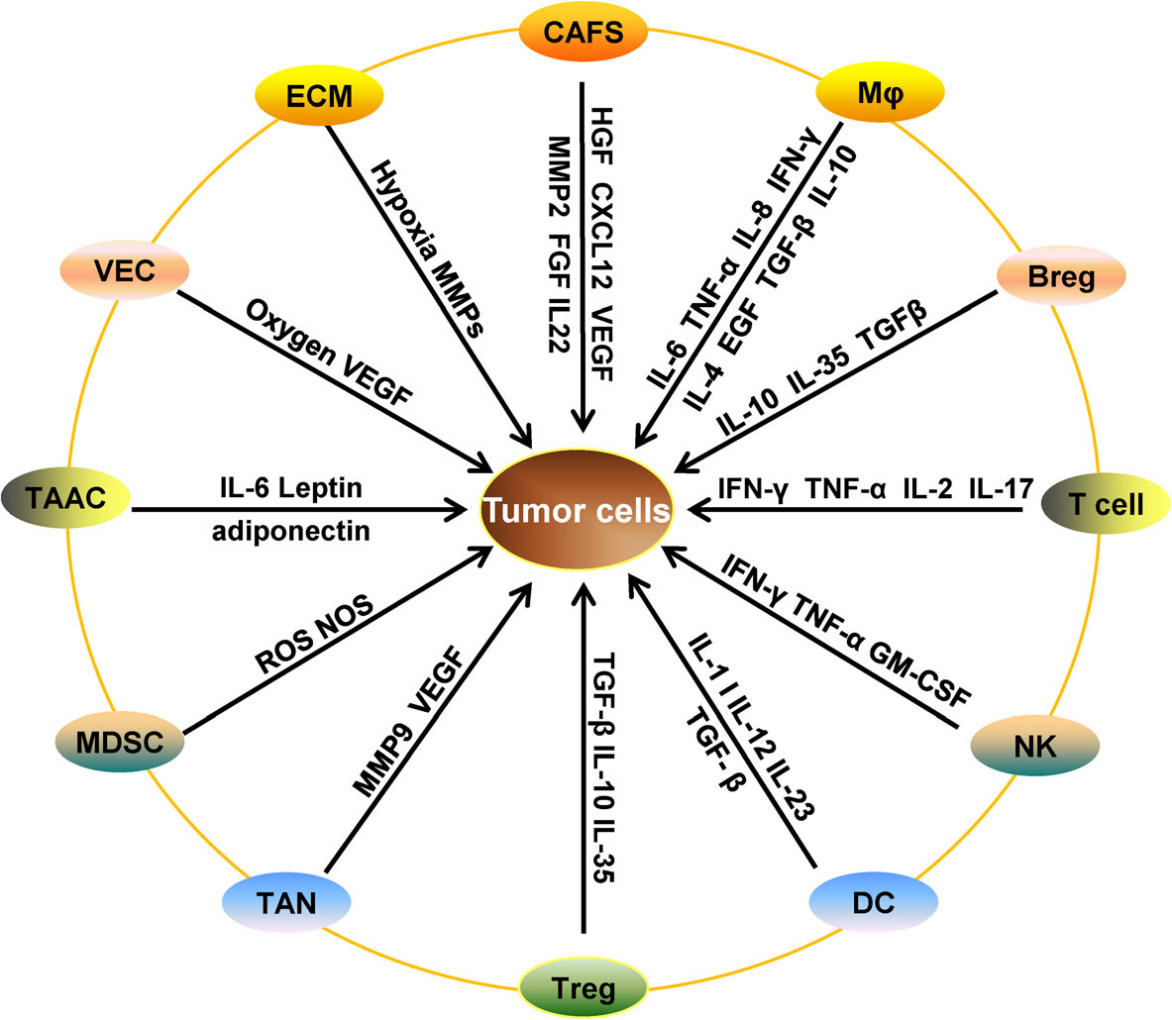
Cell growth factors and cytokines secreted in the tumor microenvironment. Abbreviations: CAFS, cancer-associated fibroblasts; Mφ, macrophage; Breg: B regulatory cell; T cells, T lymphocytes; NK, natural killer cell; DC, dendritic cells; TAN, tumor-associated neutrophil; MDSC, myeloid-derived suppressor cell; TAAC, tumor-associated adipose cells; VEC, vascular endothelial cell; ECM, extracellular matrix

A tumor is not simply a cell mass composed of malignant cells but is actually composed of a large number of non-transformed cells recruited by malignant cells, eventually forming a complex structure composed of both malignant cells and non-transformed cells, and their interaction forms the tumor microenvironment [19–24]. The tumor microenvironment consists mainly of vasculature, extracellular matrix (ECM) [25, 26], and other non-malignant cells surrounding the tumor, as well as a complex signaling molecule network that sustains the internal connections of the microenvironment, including growth factors, cytokines, chemokines, and exosomes [27, 28] (Fig. 1). In recent years, with the development of biological technology, different types of cells were identified in the microenvironment, including stromal cells, fibroblasts, fat cells, vascular endothelial cells, and immune cells such as T lymphocytes, B lymphocytes, NK cells, tumor-associated macrophages, and so on [27]. Most of these cells can secrete cytokines and play a role in promoting or inhibiting tumors. Among them, mesenchymal cells and fibroblasts can secrete growth factors such as hepatocyte growth factor, fibroblast growth factor, vascular endothelial growth factor (VEGF), metal secretory proteins MMP2 and CXCL12, and chemokines in the tumor microenvironment. These cytokines not only promote the growth and survival of malignant tumor cells but also their invasion and migration [29, 30]. Vascular endothelial cells produce blood vessels that supply oxygen to tumor cells and carry away metabolic waste. However, the blood vessels generated inside the tumor are incomplete and have weak function; thus, new blood vessels need to be generated constantly, which further complicates the internal blood vessel network [31]. The adipose tissue is an important component of the tumor microenvironment, which can provide a hypoxic and inflammatory microenvironment for tumors [32]. In particular, interleukin (IL)-6, leptin, and adiponectin secreted by fat cells play an important role in tumor growth [33].

Immune cells are the most important defensive weapon of the human body. The immune system is composed of various immune cells to protect against invading or infectious pathogens and to eliminate damaged or cancerous cells [34, 35]. The immune cells located in the tumor microenvironment comprise T cells, Tregs, B regulatory cells (Bregs), NK cells, DCs, MDSCs, and macrophages, among others. Tumor-infiltrating T lymphocytes are important effector cells in the immune system and can be divided into CD4+ T cells (helper T cells) and CD8+ T cells (cytotoxic T cells). These cells can secrete anti-tumor cytokines such as interferon (IFN)-γ, tumor necrosis factor (TNF)-α, IL-17, and IL-2. Tregs and Bregs are immunosuppressive cells in the immune system that secrete IL-10, IL-35, and transforming growth factor (TGF)-β to suppress the immune response of T lymphocytes, and thus prevent damage from the excessive activation of T cells. NK cells account for approximately 10% of the peripheral lymphocytes and are widely distributed in the peripheral blood, lymph nodes, spleen, and bone marrow, but can also migrate to an inflammatory site under the induction of chemokines. The main role of NK cells is to exert cytotoxicity. After activation, NK cells can secrete IFN-γ, TNF-α, and granulocyte macrophage-colony-stimulating factor (GM-CSF) to exert anti-tumor effects [36]. DCs can express immune co-stimulatory molecules as well as initial inflammatory factors to promote Th1 and cytotoxicity, including IL-1, IL-12, and IL-23 [37]. MDSCs are also a class of immunosuppressive cells that can up-regulate the immunosuppressive products nitric oxide synthase and reactive oxygen species to inhibit the activity of immune cytotoxic T cells [38]. Neutrophils are mainly abundant in the peripheral blood, and can produce a large number of proteases and growth factors such as MMP9 and VEGF, contributing to promoting the growth and metastasis of tumor cells [39]. Macrophages can be divided into M1 type and M2 type macrophages: M1-type macrophages have anti-tumor characteristics and secrete pro-inflammatory factors such as IL-6, IL-8, IL-1β, IFN-γ, and TNF-α, whereas M2-type macrophages have tumor-promoting properties and secrete anti-inflammatory factors such as IL-10, IL-4, epithelial growth factor (EGF), TGF-β, and IL-19, thereby playing an important role in promoting tumor cell proliferation and migration [40, 41].

The ECM consists of the basement membrane and intercellular mass, which is an important barrier for tumor metastasis. The ECM contains a large number of growth factors, cytokines, and various metalloproteinases secreted by tumor cells and other cells in the microenvironment, along with various acidic substances produced by tumor metabolism to maintain the weak acidic environment of tumors, which could induce the tumor epithelial-mesenchymal transition (EMT) and facilitate a low-oxygen environment. Indeed, owing to the rapid cell growth of tumors and the insufficiency of tumor angiogenesis, a hypoxic environment is a common characteristic of the tumor microenvironment. In addition, exosomes carrying non-coding RNAs are another important component of the tumor microenvironment, which contribute to the growth and migration of tumor cells [42, 43].

Since the PD-L1/PD-1 signaling pathway is one of the important pathways of tumor immune escape, regulating the expression level of PD-L1 could help to manipulate these components of the tumor microenvironment through inhibition of the activation of T cells so as to eliminate the immune surveillance of the tumor microenvironment and prevent tumors from achieving immune evasion.

## Effect of the PD-L1/PD*-*1 signaling pathway in immune cells

PD-1 is a specific receptor of PD-L1. After binding, PD-1 can inhibit the activation of lymphocytes, reduce the secretion of lymphocyte cytokines, and thus promote the apoptosis of lymphocytes. PD-1 protein is a type I transmembrane protein with a molecular weight of 55–60 kDa, composed of an extracellular IgV-like domain, hydrophobic transmembrane region, and intracellular region. The C-terminal and N-terminal amino acid residues of the intracellular domain of PD-1 have two independent phosphorylation sites: the immunoreceptor tyrosine-based inhibitory motif (ITIM) and the immunoreceptors tyrosine-based switch motif (ITSM) [44]. The ITSM is an important structural site for PD-1 to exert its biological function: when PD-1 binds to PD-L1, the ITSM region will be phosphorylated to activate a series of intracellular signaling pathways and achieve efficient immune inhibition. However, the specific mechanism of PD-1 activation differs between T and B lymphocytes.

### T cells

**Fig. 2:**
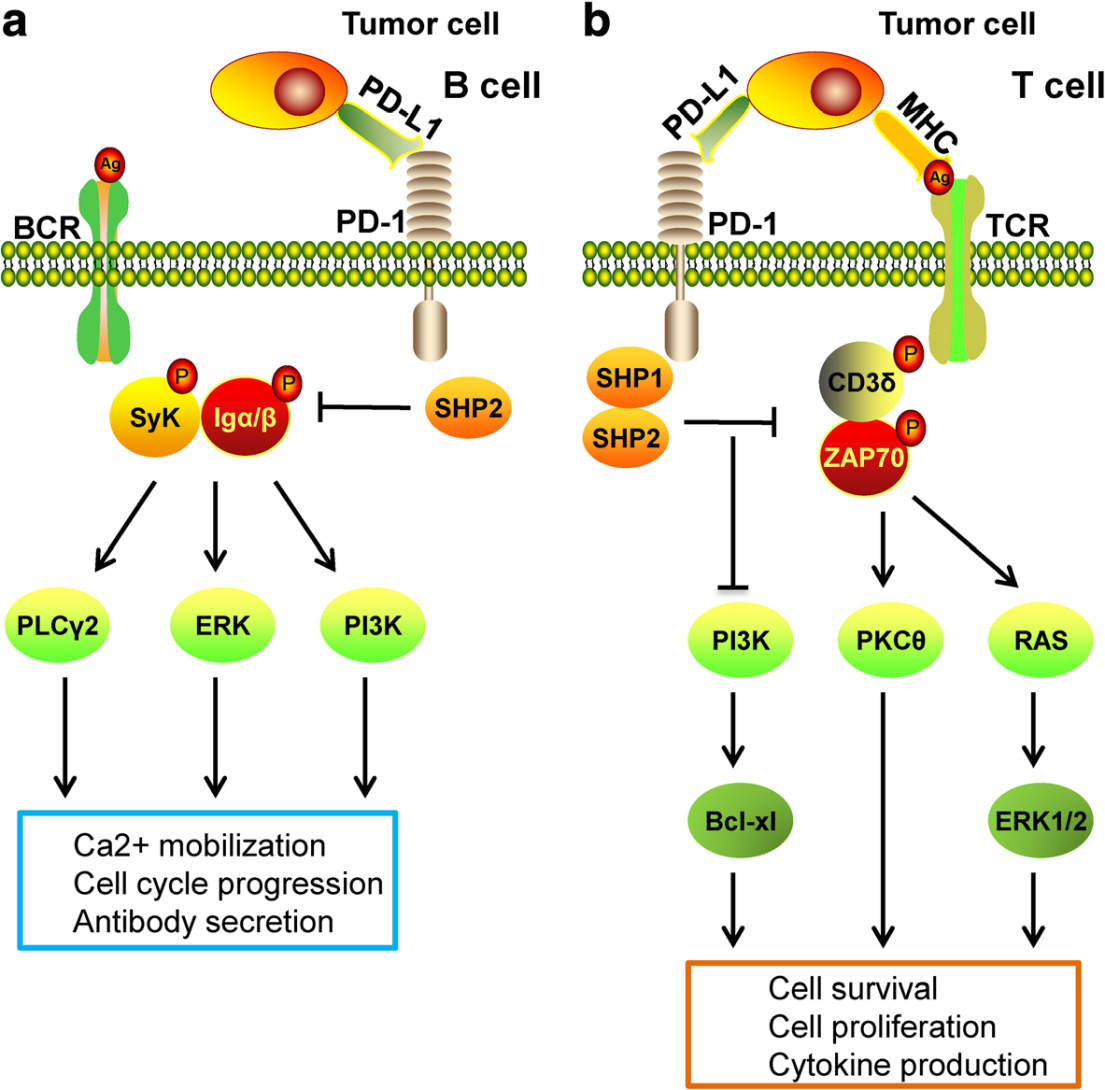
PD-1 signaling in B cells and T cells. **a** In B cells, upon PD-1 activation, SHP-2 is recruited to the C-terminal of PD-1 and dephosphorylates downstream members of the BCR pathway (e.g., SyK, Igα/β), thereby disrupting the normal BCR response as well as inhibiting PLCγ2, ERK, and PI3K signaling. This PD-1 activation consequently reduces the stability of the immunological synapse as well as B cell cycle arrest and causes disorder of Ca^2+^ mobilization. **b** In T cells, when PD-1 combines with PD-L1, SHP-1/2 are recruited to the C-terminal of PD-1 immediately and dephosphorylate key signal transducers, including the ZAP70, CD3δ, and PI3K pathways, thus suppressing TCR-mediated cell proliferation and cytokine production

The classical model of the T cell-mediated immune response during pathogen invasion involves the regulation of two signals. The first signal is the combination of the pathogen-derived peptide antigen and the major histocompatibility complex on the surface of an antigen-presenting cell (APC), which transmits signals to T cells that are identified by antigen-specific receptors. However, the first signal presented by the APC is not sufficient to activate the T cell immune response, and a second signal is required so that the immunostimulatory molecule ligand expressed by the APC interacts with the T cell receptor (TCR). Through this interaction, the signal of activation or inhibition is transmitted to T cells to regulate the immune response. Therefore, the immune co-stimulating molecule in the second signal is the key molecule of the T cell immune response, which plays an important role in T cell activation, tolerance, and apoptosis [45]. PD-L1/PD-1 can inhibit the TCR-mediated activation of T cells. The specific mechanism is as follows. When the TCR signal is activated, the ligand-bound PD-1 intracellular tyrosine is phosphorylated and then activated. PD-1 then recruits SHP-1 and SHP-2 to the C-terminal ITSM, which can dephosphorylate the TCR activation signals CD-3ζ and ZAP70, leading to downstream PI3K/AKT signaling inhibition. PI3K/AKT inactivation downregulates the expression of the cell survival gene Bcl-xl and promotes T lymphocyte apoptosis, while simultaneously inhibiting the secretion of cytokines by T lymphocytes [46]. This mechanism differs from the activation of AKT by CTLA4, in which PD-1 binds to the p85α subunit and p110σ subunit of PI3K in the TCR molecular cluster to activate early T cells, thereby inhibiting the phosphorylation of PI3K. PD-1 has also been suggested to activate PTEN, thereby inhibiting TCR-mediated PI3K/AKT activation [47, 48]. In addition, PD-1 can inhibit activation of the RAS-ERK1/2 signaling pathway to consequently inhibit the proliferation of T lymphocytes; alternatively, PD-1 can inhibit the activation of PKCδ and reduce the secretion level of IL-2 by T cells [49, 50] (Fig. 2b).

### B cells

PD-1 can also inhibit the activation of B cells. To understand the mechanism, researchers constructed a chimeric molecule composed of the extracellular region of the IgG Fc IIB receptor and the intracellular region of PD-1. After expressing this molecule in B lymphocyte strains, they found that PD-1 prevented B cell receptor (BCR)-mediated growth inhibition, and also inhibited the intracellular transport of Ca^2+^ and the tyrosine phosphorylation of some effector molecules [51]. These effects occurred because when the ligand-bound PD-1 is linked to BCR, the two tyrosines on the PD-1 ITSM are phosphorylated so that SHP-2 molecules are recruited to the C-terminus of PD-1 and phosphorylated. Phosphorylated SHP-2 can then dephosphorylate BCR signaling molecules such as SyK and Igα/β, leading to dephosphorylation of the downstream molecules PI3K, PLCγ2, and ERK, resulting in acute Ca^2+^ disorder and long-term growth stagnation. In addition, the expression of PD-1 can weaken the immune response of B lymphocytes to antigens [52]. Activation of PD-1 also inhibits the secretion of antibodies by B cells in the presence of type 2 antigen stimulation [53, 54] (Fig. 2a).

Therefore, many factors in the tumor microenvironment can induce PD-L1 expression and promote immune escape, which will be discussed in detail in the following section.

## Regulation network of PD-1/PD-L1 in the tumor microenvironment

### Regulation of PD-1

**Fig. 3:**
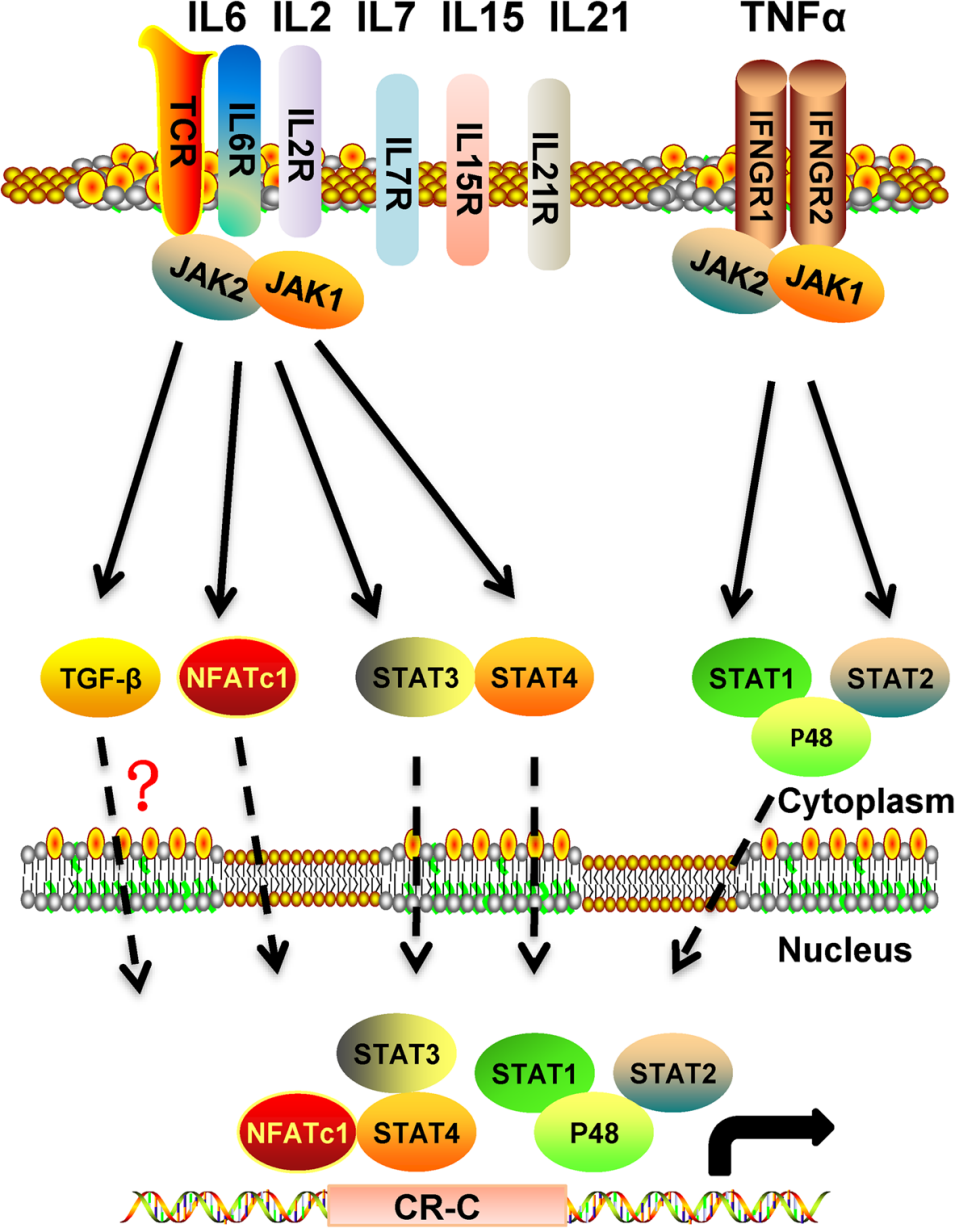
The regulation network of PD-1 in the tumor microenvironment

As an immunosuppressive molecule, PD-1 can inhibit the activation of T lymphocytes and promote T lymphocytes apoptosis. PD-1 is also affected by the tumor environment (Fig. 3). The expression of PD-1 is strictly regulated in T lymphocytes, with low or even undetectable expression in naïve T lymphocytes, but is rapidly induced when the TCR signal is activated [55]. TGF-β plays an important role in in this process. When TGF-β is blocked, the TCR activation-induced PD-1 can be inhibited significantly [56]. Moreover, when stimulated by an antigen (e.g., anti-CD3), CD4+ T lymphocytes, CD8+ T cells, NK T cells, and monocytes are all induced to express PD-1 on the cell membrane [57]. This may be related to the body’s own protective mechanism, which can inhibit the excessive activation of immune cells. Upon TCR activation, the influx of calcium ions into cells will activate NFATc1, when then translocates to the nucleus where it can bind to the 5′-terminal region of the PD-1 promoter, thereby increasing the transcription level of the PD-1 gene [58]. In addition, IL-12 and IL-6 can also induce PD-1 upon TCR activation by changing the chromatin structure of the PD-1 gene and enhancing the transcription of PD-1 by activating STAT3/STAT4. However, this effect cannot be achieved by IL-6 or IL-12 alone [59], and requires the proximal cis-element CRC of the PD-1 promoter, along with the transcription factors FOXO1 and NF-κB [60]. Other factors in the tumor microenvironment can also regulate the expression of PD-1. IL-7, IL-15, and IL-21 can induce PD-1 expression in peripheral T lymphocytes, although up-regulated PD-1 does not affect the expansion and survival of T cells by these cytokines, but rather inhibits the secretion of cytokines in T lymphocytes [61]. In macrophages, IFN-α can also regulate the expression of PD-1 by activating the JAK/STAT signaling pathway, which can form the P48/STAT1/STAT2 complex that binds to the ISRE binding site on the PD-1 promoter, thereby enhancing PD-1 transcription [62]. In addition, in mouse T lymphocytes, IFN-α can also act synergistically with TCR signals to regulate the expression of PD-1, and generates strong inhibition feedback signals for T lymphocyte-mediated immune responses [63]. In addition, the inflammatory factors TNF-α and IL-6 can also neutralize the growth inhibition of PD-1 on T lymphocytes in osteoarthritis, which is mainly achieved by inducing the secretion of soluble PD-1 to interfere with the interaction of PD-1 and PD-L1 [64]. To date, few studies have focused on the regulation of PD-1, and thus the detailed regulation mechanism is not very clear. With the development and deepening of immunotherapy, it is believed that more extensive and in-depth research will be conducted on the regulation mechanism of PD-1 in the future.

### Regulation of PD-L1

**Fig. 4:**
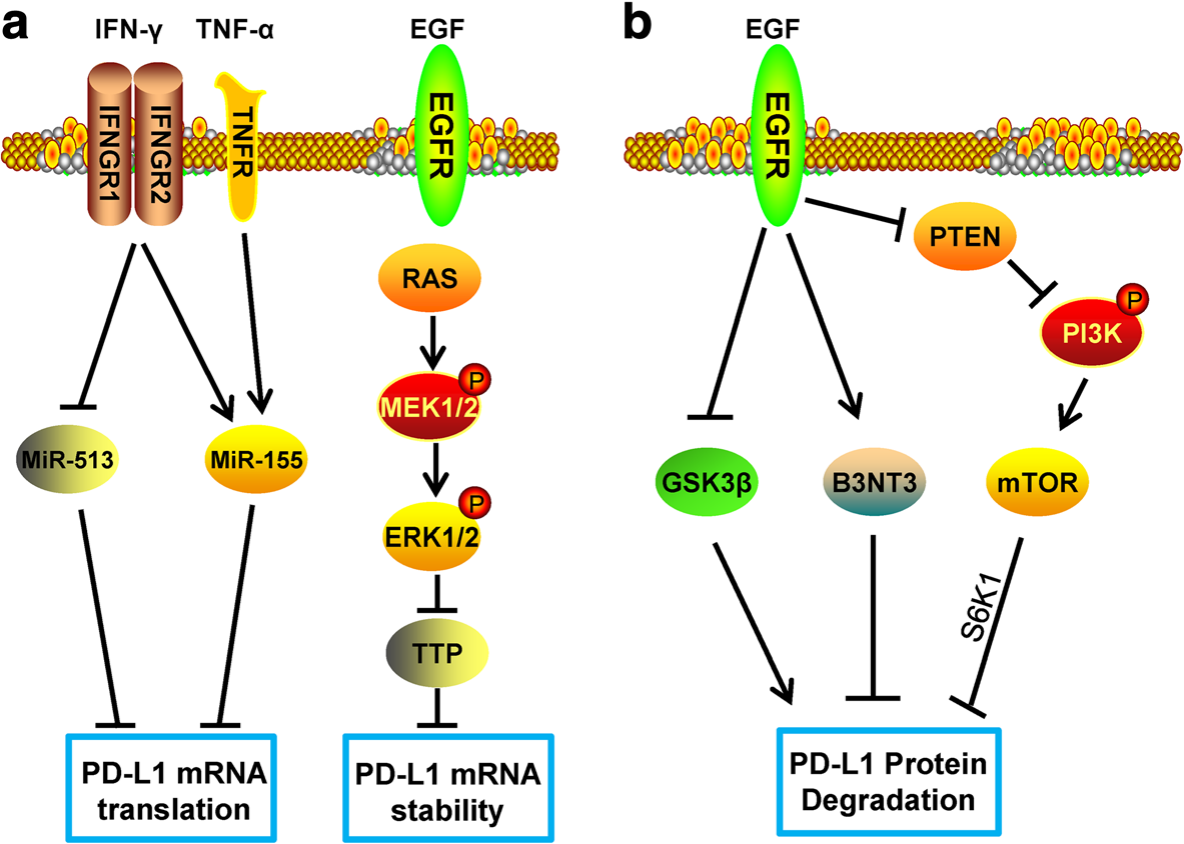
Epigenetic modification of PD-L1 by the tumor microenvironment. **a** IFN-γ can regulate the translation of PD-L1 mRNA via upregulating miR-155 or downregulating miR-513, and EGF can enhance the mRNA stability via the RAS-ERK1/2-TPP pathway. **b** EGF can reduce PD-L1 degradation via upregulating B3NT3 or downregulating GSK3β; alternatively, EGF can enhance PD-L1 protein stability via the PTEN/PI3K/mTOR /S6K1 pathway

**Fig. 5:**
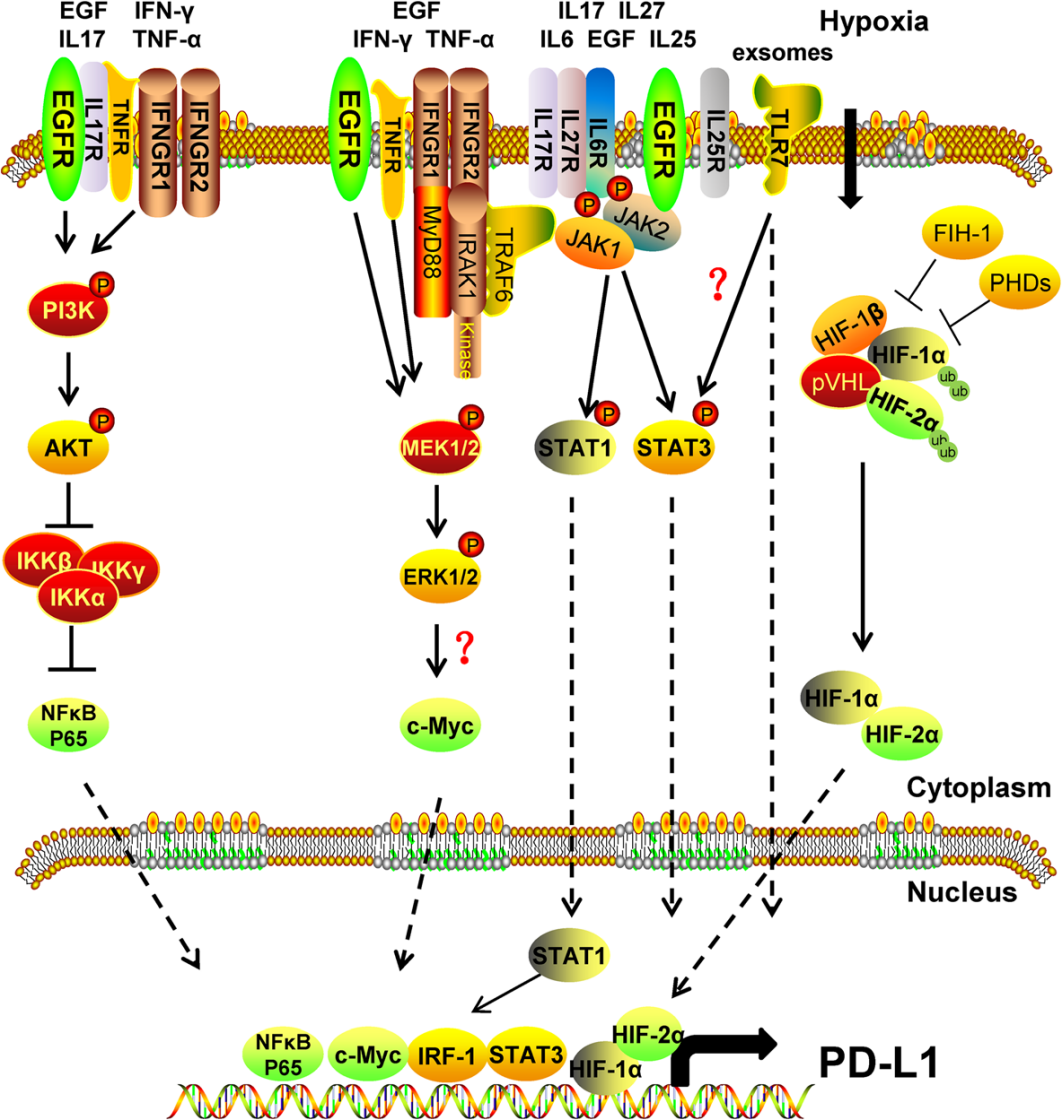
Transcription regulation network of PD-L1 in the tumor microenvironment

In addition to being widely expressed on the surface of T lymphocytes, B lymphocytes, DCs, and macrophages, high expression of PD-L1 is also found on the surface of many tumor cells, causing T cell exhaustion and immune tolerance, leading to immune escape [65]. As summarized above, a variety of cytokines and exosomes in the tumor microenvironment [66–68] can induce the expression of PD-L1, enhance the PD-L1/PD-1 signal to inhibit CTL activation in the tumor microenvironment, and thereby promote tumor escape. The mechanism of induction of PD-L1 by these cytokines and exosomes is schematically summarized in Figs. 4 and 5. Here, we focus on some of the key players of PD-L1 regulation: IFN-γ, TNF-a, cell growth factors, hypoxia, and exosomes.

### IFN-γ

IFN is a bioactive glycoprotein that is secreted by cells when they are infected by viruses, with antiviral, antibacterial, antitumor, and immune regulation functions [69]. IFN-γ belongs to the type II IFN family, which is mainly secreted by CD8+ T lymphocytes, NK cells, and macrophages. When two IFN-γ molecules interact with IFNGR1/IFNGR2, a biologically active tetrameric complex can be formed, which plays an important role in both innate and adaptive immunity [70]. IFN-γ has long been considered an anti-inflammatory cytokine that plays an important role in antiviral and bacterial infections as well as anti-tumor adjuvant therapy [71]. However, in recent years, accumulating evidence has indicated that IFN-γ does not show complete anti-tumor efficacy, and in some cases, can also actually promote tumor growth and resist tumor immunological monitoring [72]. For example, adjuvant treatment of IFN-γ to melanoma patients had to be terminated prematurely because these patients had a worse outcome than those who did not receive the treatment [73]. In another trial, the total survival time of patients with advanced ovarian cancer that received a combined regimen with IFN-γ and carboplatin/paclitaxel was significantly shorter than that of patients treated with carboplatin/paclitaxel alone [74]. These results demonstrated that IFN-γ therapy not only is ineffective at inhibiting the tumor but also promotes the growth of the tumor in certain conditions. The poorer prognosis of patients under IFN-γ regimens suggests that IFN-γ may have a positive regulating effect on certain tumor-promoting factors. With increased research focus on immune checkpoints in recent years, IFN-γ was shown to induce the expression of PD-L1 and thus promote the escape of tumor cells from the body’s immune surveillance by protecting tumor cells from specific T lymphocytes, and ultimately promoting tumor progression [75]. When IFN-γ binds to its receptor, the dimer formed changes the conformation of the receptor, and the allosteric complex narrows the distance between JAK1/JAK2 and IFNGR1/2, prompting the autophosphorylation and activation of JAK2 [76].

IFN-γ has multiple ways of inducing the expression of PD-L1, which is related to the type of tumor. In gastric cancer, IFN-γ induces PD-L1 expression via the JAK2/STAT1/IFR-1 signaling pathway [77, 78]. However, in lung cancer, IFN-γ induces PD-L1 expression through the JAK/STAT3 and PI3K-AKT signaling pathways, leading to immune escape [79]. Similar results have been obtained in dermal fibroblasts. In addition, the phosphorylation of MAPK and PI3K in dermal fibroblasts was found to result in the release of NF-κB, which enters the nucleus and directly binds to the PD-L1 promoter to activate its transcription [80]. However, in myeloma cells, the expression of PD-L1 induced by IFN-γ was shown to mainly occur through the MEK/ERK signaling pathway, with only a weak effect of the JAK-STAT signaling pathway and no influence of the PI3K/AKT and NF-κB signaling pathways. In addition, the inducible expression of PD-L1 by IFN-γ also relies on Toll-like receptor (TLR) signaling, as inhibiting the expression of MyD88 and TRAF6 will directly block the expression of PD-L1 induced by IFN-γ [81]. Furthermore, IFN-γ can modulate the expression of PD-L1 via microRNA (miRNA) regulation. PD-L1 is the target gene of miRNA-155 and miRNA-513, and IFN-γ can inhibit the expression of miRNA-513 to relieve the translation inhibition of PD-L1, thereby enhancing the expression of PD-L1 protein [82]. However, IFN-γ can also increase the expression level of miRNA-155, which may contribute to a feedback inhibition loop of PD-L1 expression induced by IFN-γ [83].

Overall, these findings demonstrate that the effect of IFN-γ on a tumor is complex. However, further study of the regulation mechanism of PD-L1 expression by IFN-γ will be helpful to understand the promotion of tumorigenesis by IFN-γ, and to find a new method to reduce the side effects (i.e., tumor-promoting effects) in the treatment of tumors with IFN-γ therapy.

### TNF-α

TNF was originally identified as a direct contributor to tumor hemorrhagic necrosis. However, subsequent studies indicated that TNF-α is an important inflammatory factor in addition to its effects of killing tumor cells. TNF-α is produced by kinase-activated macrophages, which bind to the receptor of specific homologous trimers on the cell membrane. By activating the caspase protease, JNK, and transcription factor NF-κB signaling pathways, TNF-α can induce inflammation, and promote cell growth, differentiation, and apoptosis. Recent studies have found that TNF-α also induces the expression of PD-L1. In a mouse model, the expression of PD-L1 increased during the maturation of monocytes to macrophages, and reached its peak when monocytes differentiated into macrophages. Moreover, these effects were found to be independent of IFN-γ expression. Although an abundance of IFN-γ and TNF-α will be produced during the differentiation of monocytes into macrophages, in vitro experiments confirmed that certain cytokines would be produced when tumor cells are co-cultured with monocytes, which could promote the secretion of TNF-α by monocytes along with the expression of PD-L1 on the surface of monocytes [84]. The expression of PD-L1 is deficient in the monocytes of patients with systemic lupus erythematosus, but can be reconstituted by the exogenous addition of TNF-α [85]. In addition, HIV infection often leads to increased expression of the co-immunosuppressive molecule PD-L1, mainly due to the role of Tat protein. In DCs, induction of Tat protein on PD-L1 was found to be closely related to TNF-α [86]. TNF-α can up-regulate the mRNA and protein levels of PD-L1, which is mainly completed by activating the NF-κB and ERK1/2 signaling pathways [87]. In addition, TNF-α can also modulate the expression of PD-L1 through miRNA-155 [83]. As an inflammatory cytokine, TNF-α plays an important role in activating inflammatory cells, killing pathogens, participating in tissue repair, and inducing angiogenesis and connective tissue formation. However, recent studies have shown that TNF-α can also induce the expression of the immunosuppressive molecule PD-L1 to promote immune escape. Therefore, the role of TNF-α in tumors is still unclear and needs further study.

### ILs

ILs are types of cytokines produced by various cells that play an important role in the maturation, activation, proliferation, and immunomodulation of immune cells. In addition, ILs are involved in various physiological and pathological responses. For example, IL-27 is a key immunosuppressive cytokine that can induce the expression of PD-L1 in initial T cells, which is a STAT1-dependent event. In vivo experiments showed that T cells expressing a TCR transgene and IL-27-induced PD-L1 could inhibit the differentiation of Th17 cells and avoid the development of severe autoimmune encephalomyelitis [88]. Other than T cells, induced expression of PD-L1 by IL-27 can be found in ovarian cancer cells, prostate cancer cells, and non-small cell lung cancer cells [89]. As pro-inflammatory cytokines, IL-6 and IL-17 also mediate the expression of PD-L1 in the tumor microenvironment. Epithelial growth factor receptor (EGFR) regulates the expression of PD-L1 and cell proliferation through the IL6/JAK/STAT3 signaling pathway [90]. Moreover, high expression of PD-L1 and low expression of NKG2D can enhance the radiotherapy tolerance of non-small cell lung cells, which is mainly achieved through the IL-6/MEK/ERK signaling pathway [91]. Further, inhibition of the IL-6 signal pathway can reduce the expression level of PD-L1 and enhance the sensitivity of cancer cells to the cytotoxicity of NK cells [92]. Although IL-10 itself does not directly induce the expression of PD-L1, after inhibiting the expression of IL-10 in the environment, the expression level of PD-L1 also decreases. This suggests that IL-10 can induce the expression of PD-L1 via certain factors present in cells [93]. IL-12 is best known for its important role in the activation of NK cells and T lymphocytes but has also been shown to induce PD-L1 expression. This inducing effect depends on activation of the NF-κB signal, which may be related to the direct induction of IFN-γ by IL-12 [94]. Similar to TNF-α, IL-17 can also induce the expression of PD-L1 through the synergistic effect of the NF-κB and ERK1/2 signaling pathways [87]. In human pluripotent interstitial cells, IL-25 can inhibit the immune response of Th17 cells via the IL-25/STAT3/PD-L1 complex [95].

### Cell growth factors

EGF is a small-molecule active polypeptide that is widely distributed in the human body, consisting of 53 amino acids. EGF can combine with its receptor EGFR and activate EGFR signaling pathways to promote cell growth. Moreover, the EGFR signaling pathway plays an important role in tumor cell migration and proliferation, and mutations of EGFR can be found in many types of tumor cells. The mutated EGFR no longer needs to be combined with EGF to have a continuous activation effect, leading to malignant proliferation and metastasis of the tumor. Recent studies have shown that EGFR signals can not only regulate the growth and invasion of tumors but also play an important role in their immune escape. In various cancers such as lung cancer, breast cancer, head and neck cancer, esophageal cancer, and salivary adenoid cystic carcinoma, EGF was found to up-regulate the expression of PD-L1. However, the specific mechanism by which EGF induces PD-L1 differs according to the cancer type [96–100]. In lung cancer and head and neck cancer, EGFR can increase the expression of PD-L1 via activating the JAK/STAT1 signaling pathway [98, 101]. In addition, PI3K/AKT and MEK/ERK are also important pathways for inducing the expression of PD-L1 in lung cancer cells with EGFR mutation [102]. MYC is an important transcription factor in tumors and is also involved in the regulation of PD-L1 by EGFR. In the EGFR-derived PD-L1 signaling pathway, inhibiting the expression of MYC can significantly reduce the expression of PD-L1 [100, 103]. In T-ALL cells, we found that MYC can be directly combined with the PD-L1 promoter to upregulate the expression of PD-L1 [104], suggesting that the EGFR pathway could up-regulate the expression of the MYC transcription factor and promote its nuclear translocation to ultimately up-regulate the expression of PD-L1. EGF not only induces the transcription of PD-L1 but also affects its protein stability and biological function. EGFR-RAS is a classical intracellular signal pathway, and RAS has been shown to regulate the mRNA stability of PD-L1. In lung cancer and colorectal cancer, RAS can enhance the stability of PD-L1 mRNA via the phosphorylation of TPP by MEK signaling, as TPP can negatively regulate the stability of PD-L1 mRNA. EGFR-RAS can also increase the protein level of PD-L1 and enhance the immune tolerance of the tumor [105]. In tumor cells, a decrease in the PTEN copy number is often accompanied by an increase in the EGFR copy number [106], and the decreased PTEN leads to activation of the PI3K/AKT signaling pathway. The activated PI3K/AKT signal can in turn stabilize the protein level of PD-L1 through mTOR/S6K1 and promote immune escape of the tumor [107]. In addition, EGF can inhibit the protein level of GSK3β in cells, while GSK3β can phosphorylate PD-L1, making it more susceptible to ubiquitination and degradation to ultimately increase the stability of PD-L1 protein. The glycosylation level of the PD-L1 protein itself also directly affects the degradation of GSK3β [97]. B3GNT3 is one of the proteins that glycosylate PD-L1. EGF can upregulate the expression of B3GNT3 to promote the glycosylation of PD-L1, which will also enhance the stability of PD-L1 protein and promote its biological function [108]. Therefore, blocking the EGFR signaling pathway, in addition to inhibiting tumor growth, can also inhibit tumor immune escape, which is undoubtedly a benefit for EGFR blockage therapy.

The TGF-β family of polypeptide signaling molecules plays an important role in regulating cell growth and differentiation. Similar to its dual role in both promoting and inhibiting cancer, TGF-β is a double-edged sword with respect to tumor immune escape. In hepatocellular carcinoma and lung cancer, TGF-β can induce the expression of PD-L1 on the surface of DCs, which depends on the activation of STAT3 [109, 110]. However, in systemic lupus erythematosus, TNF-α can induce the expression of PD-L1 in mononuclear cells, while TGF-β has the opposite effect. This is consistent with a previous report showing that TGF-β could inhibit the expression of proximal epithelial cell PD-L1 in renal tubules, although the exact mechanism is unclear [111]. TGF-β signaling pathways mainly comprise the classic TGF-β/Smads signaling pathways and the non-classical TGF-β/EGFR signaling pathways, and recent studies have shown that the influence of TGF-β on PD-L1 is almost always dependent on the latter, while the role of the former pathway in regulating the expression of PD-L1 has not been reported to date.

In addition, GM-CSF can also activate the expression of PD-L1 in neutrophils, which promotes the inhibition of their T cell immune activation and achieves immunosuppression via the JAK2/STAT3 signal pathway [112, 113].

### Hypoxia

During the occurrence and development of tumors, especially solid tumors, it is impossible to rapidly establish new blood vessels in the tumor microenvironment to match the rapid proliferation rate of tumor cells. Furthermore, the newly established endovascularization results in abnormalities in structure and function. Ultimately, the metabolism of tumor cells results in a tumor microenvironment characterized by a decrease of oxygen content, lack of nutrients, and accumulation of a large number of acidic substances, and these conditions are extremely detrimental to the growth of tumor cells [114–118]. However, in an anoxic microenvironment, tumor cells can change their metabolic mode to conduct metabolic reprogramming via changing the expression of glycolytic-related proteins such as GLUT1, GLUT3, PKM2, and LDHA to ultimately increase the uptake of glucose and the production of energy [119–122]. Moreover, the invasion and metastasis of tumor cells are promoted by altering the expression of E-cadherin, N-cadherin, Snail, and vimentin, which are all EMT markers [123, 124]. Tumor immune escape is also an important strategy for tumor cells to survive in this hypoxic microenvironment.

Hypoxia-inducible factors (HIFs) are the most important proteins for cell-induced expression in hypoxic environments. Under the action of normal oxygen, proline residues in the conserved region of HIF subunits are hydroxylated by proline hydroxylase, and hydroxylated HIF is identified and ubiquitinated by the VHL E3 ubiquitination ligase so that the ubiquitinated HIFs are rapidly degraded by the proteasome [125]. However, under hypoxic conditions, the prolinyl hydroxylase of HIF protein is inhibited, which stabilizes the protein level of HIFα, resulting in induction of the expression of its target genes to induce tumor immune escape [126]. HIF induces tumor immune escape mainly by inducing the expression of PD-L1. Under hypoxia, the expression of PD-L1 has been shown to be elevated in T lymphocytes, DCs, MDSCs, and macrophages, as well as in prostate cancer cells, breast cancer cells, and colorectal cancer cells [127, 128]. Hypoxia can increase the expression of PD-L1 and suppress the killing effect of CTL on tumor cells. The induced expression of PD-L1 by hypoxia mainly occurs at the transcriptional level. The hypoxia-induced expression of PD-L1 is achieved through HIF-1α rather than HIF-2α in breast cancer and prostate cancer [128], and similar results were obtained in MDSCs. Immunoprecipitation and luciferase reporter gene experiments confirmed that HIF-1α could activate the transcription of PD-L1 via binding to the HRE site on the PD-L1 promoter [127]. However, in clear cell renal cell carcinoma, the induced expression of PD-L1 mainly occurs through HIF-2α rather than HIF-1α [129]. This difference may be related to the cell types, because over 90% of patients with clear cell renal cell carcinoma will present with pure homozygous inactivation of VHL protein, which is the E3 ubiquitination ligase of the HIF ligase subunit. This inactivation leads to high levels of HIF protein accumulation in cells [130]. In addition, other studies have suggested indirect regulation of PD-L1 by the host HIF, because the expression levels of GLUT1 and PD-L1 show a strong correlation in a hypoxic state. However, the specific mechanism of action remains to be elucidated [131].

### Exosomes

Almost all cells release exosomes, which are extracellular vesicles with a diameter of 40–150 nm. The outer part of exosomes is a lipid bilayer structure, along with proteins, DNA, and RNA from exosome-derived cells [42]. Exosomes were previously considered to represent a form of cell waste; however, recent studies have shown that exosomes can be transmitted as signal molecules to other cells to alter their function. Exosomes have also been shown to play important roles in tumor cell growth, migration, and angiogenesis [43]. More recent studies have also shown that exosomes play an important role in immunosuppression. In chronic lymphocytic leukemia, tumor-derived exosomes can induce the tumor immunosuppressive reaction in monocytes. Monocyte activation mainly occurs through the non-coding small RNA Yh4 in exosomes through the TLR7 signaling pathway in monocytes, which promotes the secretion of cytokines by monocytes and ultimately induces the expression of PD-L1 [132]. Tumor-derived exosomes can also promote the polarization of monocytes to M2 macrophages and the expression of PD-L1 in M2-polarized macrophages through STAT3 phosphorylation, further enhancing the immunosuppressive effects [133]; however, it is not yet clear whether exosomes also activate STAT3 through TLR7 in this case. In addition, the same study showed that tumor-derived exosomes containing PD-L1 protein have strong immunosuppressive effects [134]. Since research on exosomes is still in a preliminary stage, new functions of exosomes are expected to be discovered in the future.

### Non-coding RNA

**Fig. 6:**
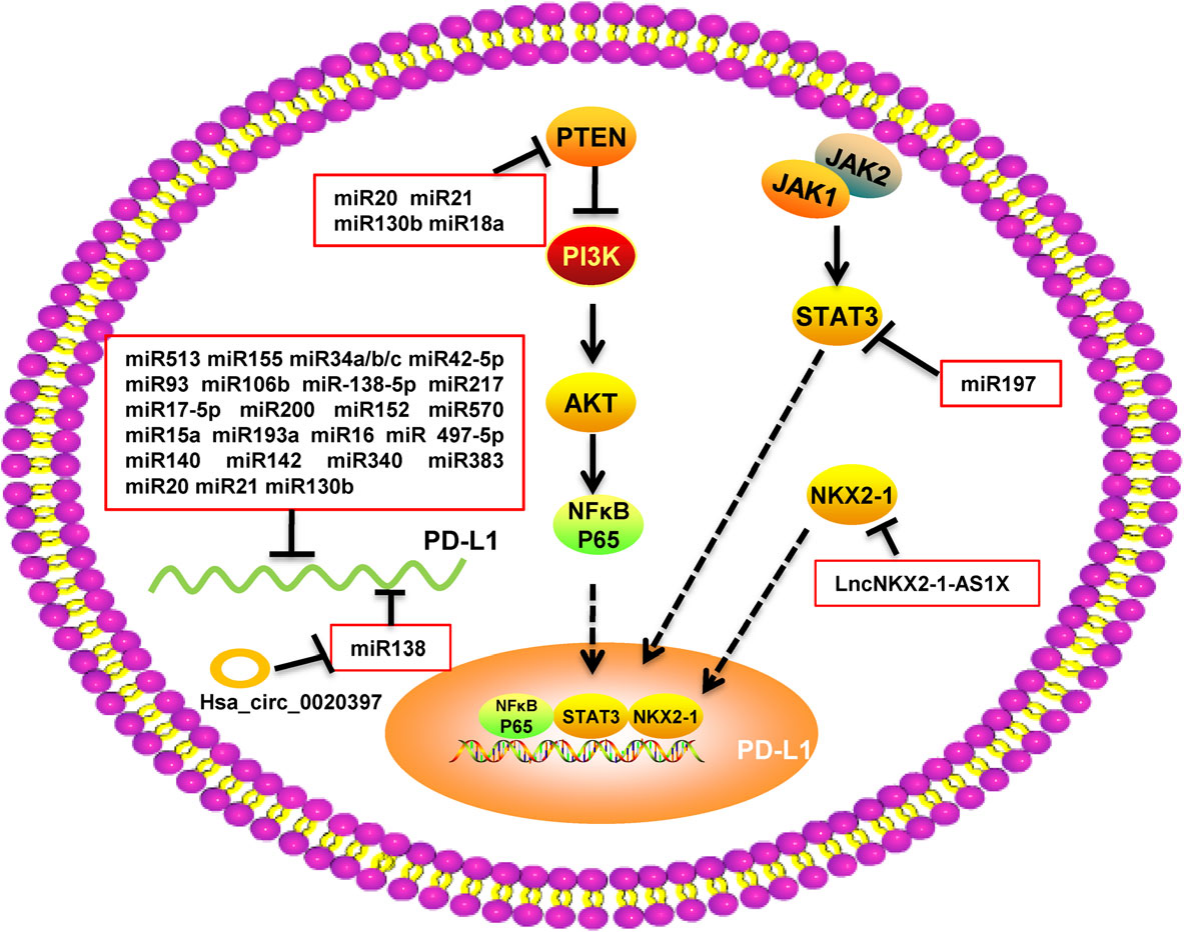
Role of non-coding RNAs in regulating PD-L1

The expression of PD-L1 in tumor cells is not only affected by the various cytokines and exosomes in the tumor microenvironment but also by various intracellular regulatory signals, including those derived from non-coding RNA (ncRNAs) [135], which transfer between cancer cells and the tumor microenvironment via exosomes and gap junctions [136]. Indeed, ncRNAs have been demonstrated to play an important role in tumor growth, metabolism, and migration, as well as in regulating the expression of PD-L1 and mediating immune escape (Fig. 6). Long non-coding RNA (lncRNA) is an endogenous RNA molecule with a length greater than 200 nt and does not encode proteins [137–142]. LncRNAs represent the majority of the sequences transcribed in the human genome, and are more abundant than protein-coding genes and small-molecule RNAs (such as miRNA), and also show more diverse and extensive patterns in regulating gene expression [143–147]. The lncRNA NKX2–1-AS1 shares some overlapping regions with NKX2–1, but there expression levels are not correlated. Instead, NKX2–1-AS1 can interact with NKX2–1 protein and interfere with its binding to the PD-L1 promoter, thereby negatively regulating the expression of PD-L1 [148]. However, there have been few studies on PD-L1 regulation by lncRNAs, and it is believed that there will be more reports on the role of lncRNAs in immune escape in the near future. MiRNAs are single-stranded small RNA molecules with a size of about 22 nt. They are generated by Dicer enzyme processing single-stranded RNA precursors with a hairpin structure of about 70–90 nt, and approximately 30–50% protein-coding genes are currently known to be regulated by miRNAs [149–151]. Mature miRNAs regulate genes in two ways: one is to bind to the target gene mRNA and promote its degradation, and the other is to inhibit the translation of target mRNA [152]. In recent years, a role of miRNAs in the regulation of PD-L1 has also been discovered. The regulatory effect of miRNA on PD-L1 occurs not only by directly binding to the PD-L1 mRNA, but also by indirectly regulating the expression of PD-L1. As the earliest miRNA identified to regulate PD-L1, miR-513 plays an important role in the IFN-induced expression of PD-L1. In cholangiocarcinoma cells, it can directly target the 3′-untranslated region (UTR) of PD-L1 and inhibit the translation of PD-L1. Overexpression of miR-513 in the induction environment can block the induction effect of IFN-γ on PD-L1 [82, 153]. In human dermal lymphoid endothelial cells and dermal fibroblasts, miR-155 induced by IFN-γ and TNF-α can also target the 3′-UTR of PD-L1 and inhibit the translation level of PD-L1 [83]. Therefore, miR-513 and miR-155 can be considered an IFNγ-induced feedback regulation loop of PD-L1. In non-small-cell lung cancer, miR-34a/b/c induced by wild-type P53 can bind to the 3′-UTR of PD-L1, promote the degradation of PD-L1 mRNA, and thereby inhibit the immune escape of non-small-cell lung cancer. In mouse models, tail intravenous injection of miR-34a contained in liposomes effectively inhibited tumor growth [154]. In addition, miR-200 can also target PD-L1 and regulate the expression of PD-L1 in non-small-cell lung cancer [155]. Several miRNAs have been shown to directly regulate the expression of PD-L1, including miR-142-5p, miR-93, and miR106b in pancreatic cancer [156, 157]; miR-138-5p in colorectal cancer [158]; miR-217 in laryngeal cancer [159]; miR-17-5p in melanoma [160]; miR-200b, miR-152, and miR-570 in gastric cancer [161, 162]; miR-15a, miR-193a, and miR-16 in malignant pleural mesothelioma [163]; miR-497-5p in clear cell renal cell carcinoma [164]; and miR-140, miR-142, miR-340, and miR-383 in cervical cancer [165]. Another way to regulate PD-L1 is through other members of the PD-L1 signaling pathway. In colorectal cancer, miR-20, miR-21, and miR130b can inhibit the expression level of PTEN, which is an upstream inhibitor of PD-L1. Therefore, these miRNAs can indirectly promote the expression of PD-L1 by inhibiting PTEN [166]. Moreover, miR-18a has similar functions. In cervical cancer, miR-18a can target PTEN, WNK2, and SOX6, leading to activation of the PI3K/AKT, MEK/ERK, and Wnt/β-catenin signaling pathways, which can promote the expression of PD-L1 [165]. In non-small cell lung cancer, miR-197 can also regulate PD-L1 by targeting CKS1B/STAT3 signaling [167].

Furthermore, recent studies have shown that circulating RNAs (circRNAs) can also regulate the expression of PD-L1. CircRNAs are a special class of non-coding RNA molecules with a closed loop structure, which is not affected by RNA exonuclease, making them more stable and less prone to degradation than other RNAs [168–171]. CircRNA molecules are rich in miRNA-binding sites, and can thus act as an miRNA sponge in cells to relieve the inhibition of miRNA on the target gene and increase its expression level. In colorectal cancer, the circRNA molecule Hsa_circ_0020397 was shown to inhibit the activity of miR-138 via an RNA sponge effect, thus promoting the expression of PD-L1 [172]. This was the first report that a circRNA can regulate the expression of PD-L1, suggesting that circRNAs might also play an important role in tumor immune escape.

## Conclusions and prospects

Immune escape mediated by the PD-L1/PD-1 signaling pathway has emerged as a hot topic in anti-tumor research and in the field of cancer translational medicine. However, targeted PD-L1/PD-1 immunotherapy has not achieved the desired effects in the treatment of various types of cancers, especially for solid tumors, with a low response rate overall. One reason for this inconsistent and poor response may be related to individual differences among patients as well as tumor heterogeneity within a single patient, given that the expression of PD-L1 varies in different tumors. In general, antagonistic drugs designed to block PD-L1/PD-1 show good effects for patients with high expression of PD-L1 or PD-1, whereas the therapeutic efficacy is poor for patients with low PD-L1/PD-1 expression, and the treatment could bring about serious side effects. Some patients that were treated with PD-L1/PD-1 antagonistic drugs actually showed rapid growth of the tumor [173]. A follow-up investigation into 131 patients that were treated with PD-1 antagonists found that 12 patients (9%) experienced deterioration, and those aged 65 and older had a higher rate of deterioration [174]. At present, there is no uniform standard for the preoperative detection of PD-L1/PD-1, making it difficult to achieve individualized treatment for a given patient with respect to the most suitable drug and dosage. In addition, the mechanism of tumor formation is very complex, and the tumor microenvironment plays an important role in the regulation of PD-L1. A variety of cytokines secreted in the tumor microenvironment can cause immunosuppression by inducing PD-L1 expression. This review summarized the main regulation mechanisms of PD-1 and PD-L1 by cytokines, exosomes, and ncRNAs in the tumor microenvironment, demonstrating the wide variation of co-molecules and pathways involved. Specifically, IFNγ can induce the expression of PD-L1 through the JAK/STAT and PI3K-AKT signaling pathways to promote tumor immune escape [79]. TNF-α can directly increase the mRNA and protein levels of PD-L1, thus promoting immune escape. IL-6 and IL-17 can also regulate PD-L1 expression by means of the JAK/STAT3 and NF-κB signaling pathways, respectively. Some growth factors such as EGF, TGF-β, and GM-CSF can also induce the expression of PD-L1 and promote the occurrence of immunosuppression. Interestingly, most of these cytokines with PD-L1-inducing abilities are inflammatory factors.

Although the effects of inflammation on tumor growth and metastasis have long been recognized, recent research has revealed that inflammatory factors can also promote the immune escape of tumors. This review further points to the potential importance of the inflammatory microenvironment for tumor immune escape. Thus, inhibiting the occurrence of inflammation in the tumor microenvironment, or inhibiting the secretion of certain inflammatory factors, may have a desirable effect for tumor treatment [175]. A study with a melanoma mouse model showed that treatment with both PD-1 inhibitors and TNF-α inhibitors had a better therapeutic effect than treatment with PD-1 inhibitors alone [176]. This indicated that the high expression of inflammatory factors in the tumor microenvironment may be one of the important factors for the poor efficacy of immunotherapy. Thus, a combination of anti-inflammatory drugs and PD-L1/PD-1 inhibitors might lead to a better treatment outcome for cancer patients.

At present, most of the drugs developed to inhibit PD-L1/PD-1 signaling pathways are antibodies; however, the latest research shows that metformin may also be effective for suppressing PD-L1/PD-1 signaling pathways. Metformin can activate AMPK [177], which can directly phosphorylate PD-L1, thus inhibiting the PD-L1 glycosylation level, resulting in PD-L1 degradation and inhibiting immune escape [178]. However, there is still much to discover about the tumor microenvironment, and the regulation network of PD-L1 by cytokines remains to be uncovered in detail. Therefore, further study of the tumor microenvironment, especially the role of inflammatory cytokines, may provide new ideas for immunotherapy and a new development direction for the effective use of PD-L1/PD-1 inhibitors in cancer treatment.

## Abbreviations

APC: Antigen-presenting cell
BCR: B cell receptor
Bregs: B regulatory cells
CTL: Cytolytic T lymphocytes
CTLA4: Cytotoxic T-lymphocyte-associated protein 4
CXCL12: C-X-C motif chemokine 12
DC: Dentritic cell
ECM: Extracellular matrix
EGF: Epithelial growth factor
EMT: Epithelial-mesenchymal transition
GM-CSF: Granulocyte macrophage-colony-stimulating factor
HIFs: Hypoxia-inducible factors
HIV: Human immunodeficiency virus
IDO1: Indoleamine-pyrrole 2,3-dioxygenase
IFN: Interferon
IFNGR: Interferon gamma receptor
IL: Interleukin
ITIM: Immunoreceptor tyrosine-based inhibitory motif
ITSM: Immunoreceptors tyrosine-based switch motif
LAG-3: Lymphocyte-activation gene 3
MDSC: Myeloid-derived suppressor cell
MMP2: Matrix metalloproteinase-2
NK: Natural killer
PD-1: Programmed death-1
PD-L1: Programmed death ligand-1
TCR: T cell receptor
TGF: Transforming growth factor
Th1: Type I T helper cell
TIM-3: T-cell immunoglobulin and mucin-domain containing-3
TNF: Tumor necrosis factor
Tregs: Regulatory T cells
VEGF: Vascular endothelial growth factor
VHL: Von Hippel–Lindau tumor suppressor
